# Network Meta-analysis to Synthesize Evidence for Decision Making in Cardiovascular Research

**DOI:** 10.5935/abc.20160052

**Published:** 2016-04

**Authors:** Leonardo Roever, Giuseppe Biondi-Zoccai

**Affiliations:** 1 Universidade Federal de Uberlândia - Departmento de Pesquisa Clínica, Uberlândia, MG - Brazil; 2 Department of Medico-Surgical Sciences and Biotechnologies, Sapienza University of Rome, Latina - Italy; 3 Department of AngioCardioNeurology, IRCCS Neuromed, Pozzilli - Italy

**Keywords:** Meta-Analysis, Evidence-Based Medicine, Review, Research, Cardiovascular Diseases, Comparative Study

## Abstract

Clinical decision-making requires synthesis of evidence from literature reviews focused on a specific theme. Evidence synthesis is performed with qualitative assessments and systematic reviews of randomized clinical trials, typically covering statistical pooling with pairwise meta-analyses. These methods include adjusted indirect comparison meta-analysis, network meta-analysis, and mixed-treatment comparison. These tools allow synthesis of evidence and comparison of effectiveness in cardiovascular research.

## Introduction

Clinical decision-making requires a balanced judgment between tasks, skills, resources, and values. This is largely beyond the reach of most researchers, and often depends on external factors that cannot be easily modulated (such as economic resources or religious beliefs).^1-5^

Systematic reviews seem to be particularly useful when combining homogenous randomized controlled trials (RCTs) and pairwise meta-analysis. Computational methods used for pairwise meta-analysis have seen momentous improvements over time, and now include patient-level approach, meta-regression, and adjustment for small study effects. The simple term network meta-analysis includes all methods of synthesis encompassing extensive evidence, indirect comparisons, mixed-treatment comparison, and multiple treatment meta-analysis.^5,6^

This article aims to summarize the key features of network meta-analysis and its potential impact on cardiovascular decision-making.

### Evidence base

#### Hierarchy of evidence

**Figure 1:**
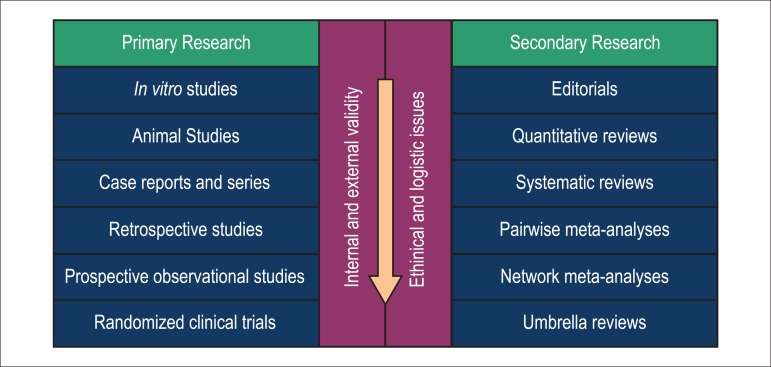
Evidence hierarchy of primary research and secondary research in cardiovascular medicine.

Evidence-based medicine emphasizes the importance of systematic research of current evidence that follows a specific hierarchy in clinical evidence, basic (bench, *in vitro*, or animal) distinctive scientific experiments, studies with healthy volunteers, case reports and patients series, cross-sectional studies, case-control studies, cohort studies, and RCTs. This hierarchy is mirrored by a hierarchy in secondary research (*i.e.*, synthesis of evidence) which includes qualitative assessments, systematic reviews, study-level pairwise meta-analyses, study-level meta-regression analyses, and finally, patient-level meta-analyses (Figure 1).^7,8^ A tertiary level of evidence and research consists of umbrella reviews, overviews of reviews, and meta-epidemiological studies.

#### From pairwise meta-analysis to network meta-analysis

Decision making is more complex than a pairwise meta-analysis since it moves from a two-dimensional to a multidimensional analytical framework. Several methods are being developed, such as adjusted indirect comparison, multitreatment meta-analysis, multi-arm meta-analysis, multivariable meta-analysis, network meta-analysis, and mixed-treatment comparison.

**Figure 2:**
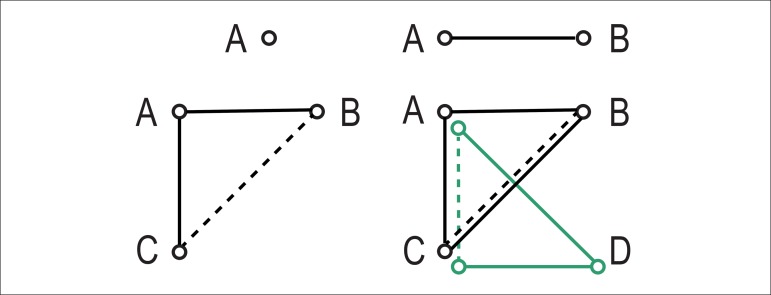
Conceptual framework moving from univariate meta-analysis (top left panel) to pairwise meta-analysis (top right panel), network meta-analysis (bottom left panel), and multivariate meta-analysis (bottom right panel). A, B, C, and D represent competing treatments for the same condition; continuous lines represent direct comparisons stemming from head-to-head randomized trials; dashed lines represent indirect comparisons; and different colors represent different endpoints of interest.

A pairwise meta-analysis can be defined as a pooled-weighted estimate of homogeneous trials comparing two treatments head to head (*e.g.*, A and B), with typically proportional weights, to study accurately the size or number of events (Figure 2). And what should we do when we have two separate sets of trials, a first comparing A versus B, and a second comparing A versus C? We perform an adjusted indirect comparison under the assumption that patients, interventions, and outcomes measured in both sets of tests are similar. And what if we then recognize that of the studies comparing A versus B and B versus C, only a few compared A versus C? Should we then discard all the information resulting from the indirect comparison, or could we explore the information and provide effect estimates, therefore, more precise and accurate of A versus C, based on both direct and indirect evidence? This is precisely what a network meta-analysis does; it combines direct and indirect evidence (where available) to provide more precise and accurate (therefore, valid both internally and externally) effect estimates to guide decision making in complex scenarios.

### Reviewing process

#### Designing and registering the review

Reviews should be designed before the data are effectively retrieved, and the evaluation protocol should be published as soon as finalized in a dedicated repository site. Several guidelines are available to design, conduct, and report a systematic peer review and network meta-analysis.^9,10^

#### Searching, selecting, abstracting, and appraising evidence

The search should be performed in various databases (MEDLINE / PubMed, Cochrane Library, Europe PubMed Central, SciELO, LILACS, Embase, and others) to appropriate evidence. The selection of the studies is an important step in any systematic review. The studies should have moderate to high methodological quality and, at the same time that they are different trials based on convenience samples, they should represent similar views on a continuum of the clinical condition and a specific management strategy or set of strategies. Finally, all studies included in the review should be assessed for internal validity.^11-14^

### Choosing the framework, package, model, and statistic

#### Choosing the statistical framework

Most biostatistical inferences are based on a frequentist approach with its defining resources: null hypothesis, alternative hypothesis, hypothesis testing, p value, and confidence interval. Therefore, they can be limited by computational problems in case of a complex evidence network. The Bayesian framework has been the dominant framework for network meta-analysis for allowing more flexible modeling and adjustment for less-than-simple evidence networks.^15-22^ Despite the arguments above, recent developments in theoretical work and improvements in computational efficiency have largely bridged the gap between frequentist and Bayesian analysis in terms of precision, accuracy, and flexibility. Thus, similar results are obtained with state-of-the-art methods, regardless of the use of frames or a frequentist-Bayesian approach.

#### Choosing the statistical package

To date, WinBUGS has been the most widely used package; it is relatively easy to command and is expressly designed for flexible Bayesian modeling and analysis. R has also been increasingly used, as it can activate WinBUGS routines, and may offer important tools for specific computations or sensitivity analyses. R can also be employed for frequentist network meta-analysis. Stata (StataCorp, College Station, TX, USA) and SAS (SAS, Cary, NC, USA) have also been adopted.^20^

#### Choosing the statistical model and between fixed and random effects

Relatively common events may best be analyzed with a binomial model, whereas uncommon events or those occurring over variable periods of time can be handled most effectively with a Poisson model.

#### Choosing the appropriate statistics

Odds ratios, relative risks, risk differences, numbers needed to treat, probabilities of being best, rankograms, and surface under the cumulative ranking curves can all be generated from a binomial model.^19,20^ Relative risks are easier to understand but suffer from a forced reduction when in the fraction of the two risks, the numerator approaches one. Both odds ratios and relative risks disregard the duration of follow-up, and hazard ratios should be preferred and considered more reliable when the follow-up is not uniform.^23,24^

The choice of risk estimator, probability of being best, rankograms, and surface under the cumulative ranking curve are now considered even more important in helping the reader identify which treatment or group of treatments should be considered most likely better than the others.^25^

#### Incorporating moderators: network meta-regression

One of the strong features of a meta-analysis is its ability to assess interaction effects with meta-regression, thus quantifying the impact of moderators or covariates in estimating the effect. Network meta-analysis is suitable for meta-regression, given its characteristics of flexible modeling.^24-26^

#### Appraising between-study heterogeneity

Evaluation of the homogeneity of similar studies is a key aspect of any systematic review. Standard methods to assess the heterogeneity between studies in pairwise meta-analysis calculations include the Cochran's Q and I-squared statistics. If the p value stemming from the Cochran's Q statistic is <0.05, then play of chance alone is an unlikely explanation for the variability in effect estimates stemming from individual studies. I-squared is interpreted as showing absent or mild between-study inconsistency if < 25%, moderate inconsistency if < 50%, and moderate to severe inconsistency for values > 50%.^4,5^

#### Appraising inconsistency between direct and indirect estimates

The most important underlying assumption of meta-analysis network is that the studies are similar enough to be considered together. Evaluation of inconsistencies in direct and indirect estimates is essential to support the validity of any network meta-analysis. Several approaches are available, but in simple terms, any meta-analysis network in which the direct and indirect estimates differ substantially should be viewed with caution or completely ignored.^17^

#### Appraising small study effects and publication bias

Small study effects may distort the overall assessment of the clinical evidence, providing estimates of inaccurate or biased effect. This is most often due to publication bias or other factors. Therefore, the assessment of small study effects is critical to support the validity of any network meta-analysis.^27^

A network meta-analysis dominated by small studies cannot be considered valid, and its results should be probably disregarded or, at best, used to generate hypotheses. Several approaches have been suggested to test for small study effects, including inspection of funnel plots after correction for subgroup summary estimates, regression testing, and the Copas method.^17,28^

#### Combining multiple effect estimates: multivariate network meta-analysis

Multivariate meta-analysis is performed on separate sets of analysis, so the reader is left with the difficult choice of considering which end point is more meaningful. One solution is to create a net composite end point (*e.g.*, nonfatal stroke, nonfatal bleeding, myocardial infarction, or death). This approach has limited benefits in terms of increased precision and forces us to consider all compounded end point components as equally important. When the results obtained with competing risks are used, there is also a risk of heterogeneous or spurious average effects (for example, when bleeding and thrombotic events are combined).

Multivariate meta-analysis is a specific application of multivariate analysis to define meta-analysis when a set of dependent variables is analyzed simultaneously, and thus when comparing different treatments, the only treatment that is most likely and more consistently capable of providing a clinical improvement may be identified. This approach is beneficial when a specific hierarchy between the different results is lacking, and when every single result, if considered isolated, has no clinical relevance to guide decision making on their own.^4^

A relevant question is whether, when assembled, end points that were only evaluated in secondary analyses may be trusted like end points that were the primary outcomes of the included studies. The risk of distortion due to reporting bias is higher in the first case, as is the risk of type I error.

#### Moving from study-level to patient-level data: individual patient network meta-analysis

Meta-analysis has always been criticized for using mostly study-level or aggregated data, and lacking originality and ecological risk. Individual patient-level meta-analysis overcomes this limitation and has many other advantages: it may improve internal validity, test subgroup hypothesis, and evaluate covariates of interest.

Network meta-analysis may be performed at both study level and patient level using an approach of one or two stages depending on the framework, package, model, and statistics of preference. While more challenging, especially in terms of logistics and cooperativeness, patient-level network meta-analysis should be considered the standard reference for any evidence synthesis effort.

#### State-of-the-art reporting of network meta-analyses

Network meta-analyses have been the focus of many standardization efforts in order to increase their robustness and validity while increasing its usability among decision makers.^5^ State-of-the-art reports should consist of explicit information about the methods, clarify the evidence network, include sound analytical methods, appraise the validity of the homogeneity and consistency assumptions, and lack substantial small study effects. Sensitivity analyses are crucial to ensure the reader of any network meta-analysis that the results are similar in statistical direction and magnitude despite different assumptions or computational methods.

### Future perspectives

#### Moving from evidence synthesis to action

The results of a network meta-analysis should be used to guide decision making, define how to best interpret the results of the evaluation and apply them in clinical practice, and to fully implement the intervention in details with the most favorable risk-benefit balance. This is best done by absolute risk estimates, numbers needed to treat, and rankograms, basing judgment on credible or confidence intervals, rather than on point estimates, while recognizing the simultaneous effect of a particular intervention on various end points.^13^ With this, when two or more interventions seem to have a similar beneficial risk-benefit profile, the one easier or cheaper to implement should be favored.

#### The future of network meta-analysis: toward accessibility and integration

The future of network meta-analysis depends on the difficult process of navigating between the Scylla of state-of-the-art processes of conducting a valid systematic review and the Charybdis of effective dissemination and successful implementation by decision makers and stakeholders. Research and clinical practice have been dominated over the past decades by simple and easy to use tools providing new solutions to complex problems. An excellent resource for clinical research methods is survival analysis using the Kaplan-Meier method, with its precise, accurate, and robust results in everyday research, despite its application in a multitude of very different and sometimes difficult contexts.

In the future, network meta-analysis and synthesis evidence will be possible with the concomitant application of simple, yet robust packages to perform network meta-analysis on various platforms such as tablets and smartphones, and the creation of intelligent trial repositories that can upload automatically the information obtained through individual data in a kind of cumulative network meta-analysis. No individual meta-analysis should be seen as the end, but rather, as a tool to provide a distilled and purified form of the available evidence to guide more accurately the clinical practice.

## Conclusions

Decision making in cardiovascular practice is often based on complex, yet incomplete evidence. Network meta-analysis represents a uniquely versatile and powerful tool to improve cardiovascular decision making.
